# Drying Techniques and Storage: Do They Affect the Nutritional Value of Bee-Collected Pollen?

**DOI:** 10.3390/molecules25214925

**Published:** 2020-10-24

**Authors:** Antonella Castagna, Giovanni Benelli, Giuseppe Conte, Cristina Sgherri, Francesca Signorini, Cristiano Nicolella, Annamaria Ranieri, Angelo Canale

**Affiliations:** 1 Department of Agriculture, Food and Environment, University of Pisa, Via del Borghetto 80, 56124 Pisa, Italy; antonella.castagna@unipi.it (A.C.); giuseppe.conte@unipi.it (G.C.); cristina.sgherri@unipi.it (C.S.); anna.maria.ranieri@unipi.it (A.R.); angelo.canale@unipi.it (A.C.); 2 Interdepartmental Research Center “Nutraceuticals and Food for Health”, University of Pisa, 56124 Pisa, Italy; 3 Consorzio Polo Tecnologico Magona, via Magona snc, Cecina, 57023 Livorno, Italy; f.signorini@polomagona.it; 4 Dipartimento di Ingegneria Civile e Industriale, Università di Pisa, Largo Lucio Lazzarino 2, 56122 Pisa, Italy; cristiano.nicolella@unipi.it

**Keywords:** amino acids, beekeeping, flavonoids, honeybees, nutraceuticals, proline, rutin

## Abstract

In this study, the effect of different drying processes (freeze-drying (FD), microwave-assisted drying (MWD) and classic hot air drying (HAD)) on the polyphenols, flavonoids, and amino acids content was investigated on bee-collected chestnut, willow and ivy pollen for human consumption. Furthermore, the pollen chemical properties were monitored after three and six months of storage, and then analyzed using a multivariate approach. Chestnut pollen was the richest source of polyphenols, flavonoids, and rutin, while ivy pollen contained the highest amount of total and free amino acids, and total and free proline. Drying and storage affected pollen chemical composition with species-dependent effects. MWD allowed the best retention of flavonoids in chestnut pollen for up to six months of storage. All drying techniques led to a depletion of flavonoids in willow pollen; however, MWD ensured the highest flavonoids content after six months. FD and MWD did not lead to flavonoids depletion in ivy pollen during storage. Additionally, storage did not affect the rutin content, which was highest in FD willow samples after six months. Notably, both FD and MWD techniques are efficient in preserving amino acids-related quality of bee pollen up to six months of storage.

## 1. Introduction

Boosting food production and quality under a growing population scenario is a major Sustainable Development Goal of the United Nations [1]. In this framework, a better understanding of how food processing and storage affects its nutraceutical composition and shelf life is an interesting area of research [2]. The physicochemical properties of food can be strongly impacted by many external factors, including, but not limited to, temperature, humidity, pressure, and various processing treatments, such as microwave-assisted drying (MWD), freeze-drying (FD), and sonication [3,4].

Beekeeping products include a rather wide range of foods and nutraceuticals [5,6]. Among these products, honeybee-collected pollen has received relatively little attention [7,8] when compared to honey, royal jelly, and propolis. However, bee-collected pollen is an important source of essential amino acids, antioxidants (with special reference to flavonoids, [9,10,11]), minerals, vitamins, and lipids [12,13,14,15,16,17]. 

The physicochemical properties of bee-collected pollen can be affected both by processing techniques [7,11,18,19,20,21] and storage conditions [22,23,24,25,26,27]. Pollen freshly collected by honeybees contains from 15% to 30% (*w*/*w*) of water. Therefore, it needs to be promptly processed to boost its physicochemical stability, avoiding microbial development [28,29]. Classic drying methods are scarcely standardized, mainly relying on pollen’s exposure to hot air flow (hot air drying, HAD), which may lead to the production of Maillard’s compounds as well as to negative effects on antioxidants and lipids content [7,30]. To solve these problems, recent research has proposed novel methods operating at lower temperatures, i.e., FD and MWD, to reduce the water content in bee-collected pollen. Both methods have been successfully employed to reduce the water content of freshly collected pollen, showing little impact on flavonoids, complex B vitamins, and/or unsaturated lipids (including vitamin E) [7,11,16,18].

The nutritional value of other pollens commonly collected by bees in the Mediterranean areas, such as ivy (*Hedera helix*), has been scantly investigated [31]. Additionally, limited information is available on how storage conditions may affect the nutritional value of bee-collected pollen for human consumption [23,24,25,27].

In this study, three main questions were addressed: (*i*) what is the content of polyphenols, flavonoids, and amino acids in ivy, chestnut, and willow pollen collected by honeybees? (*ii*) Do HAD, FD, and MWD affect the chemical properties of these pollens for human consumption? (*iii*) What is the impact of prolonged storage (up to six months) on the chemical properties of bee-collected pollen? To address these questions, variations in free and total amino acids and proline as well as in their ratios and the level of the glycosylated flavonoid rutin (a marker of pollen commercial quality, [30] of the three bee-collected pollens were investigated in fresh and dehydrated samples immediately after the treatments and after three and six months of storage.

## 2. Results

### 2.1. Influence of Plant Species, Drying Treatment, and Shelf Life on Phenolic Compounds, Flavonoids, and Rutin 

Table 1 reports the concentration of phenolic compounds, flavonoids, rutin of chestnut, ivy, and willow pollen subjected to different dehydration treatments and stored up to six months. All the three parameters were significantly influenced by the pollen origin (i.e., tree species) as well as by the drying treatment and the shelf-life period. 

Chestnut pollen was the richest source of these bioactive compounds, followed by willow. Fresh ivy pollen contained only about 26% of the phenolics, 15% of the flavonoids, and less than 3% of rutin present in chestnut pollen. The three drying treatments influenced differently the concentration of phenolic compounds depending on the species considered. 

In chestnut pollen, the conventional drying induced a significant decrease (−6%) in the concentration of phenolic compounds at t0, while MWD increased their level (+23%) with respect to fresh pollen. At any storage time, the HAD treatment was always associated with the lowest phenolic concentration. MWD allowed the best retention of phenolic compounds during storage, though at t6 no differences were observed between FD and MWD samples.

Ivy pollen underwent a 7% increase in its phenols concentration at t0 immediately after the FD treatment, while the other dehydration methods did not affect this parameter. However, after three and six months of storage, the highest phenolic concentration was detected in microwave-dehydrated samples (+46% and +58% at t3 and t6, respectively, as compared to HAD samples at the same storage time frame). 

In willow pollen, at t0, phenolic concentration was negatively affected by all dehydration treatments particularly by HAD (−18%), which was instead able to better preserve the phenolic content after three months of storage, as compared to the other two techniques. However, at the end of the storage period, this treatment was the least effective.

As observed for phenolic compounds, flavonoid concentration was also influenced by the dehydration treatments during storage in a species-dependent way. Particularly in chestnut pollen, MWD allowed the highest conservation of flavonoids at t0 and up to six months-storage (+61% and +22%, respectively, as compared to HAD), while conventional drying was the worst one, similar to what was observed for phenolics.

FD and MWD were instead the most suitable treatments for ivy pollen. Though only the first one induced an increased flavonoid content at t0 as compared to fresh pollen (+30%), after three and six months, no difference was detected between the two treatments, which were more effective in maintaining higher flavonoid concentration than conventional HAD. No flavonoid depletion was detected on ivy pollen during storage.

Conversely, in willow pollen, all dehydration treatments depleted the flavonoid content at t0, with HAD being the worst method (−29%). At the end of the storage period, the lowest and the highest flavonoid concentrations were detected in HAD and MWD samples, respectively.

The concentration of rutin, a flavonoid recognized as a valid indicator of correct dehydration method and pollen quality, was reduced by conventional HAD (−26%) and increased by MWD (+13%) in chestnut pollen at t0. During storage, rutin amounts did not vary, and after six months, FD and HAD samples showed the highest and the lowest levels, respectively. 

Rutin concentration of ivy pollen was extremely low and was negatively affected by HAD and MWD treatments at t0 (−58% and −36%, respectively). However, at the end of the storage period, no statistical differences were evident among the three dehydration treatments. 

Differently from what was observed for flavonoids, rutin concentration of willow pollen increased immediately after dehydration treatments, mainly following conventional HAD (+76%) and MWD (+55%). However, during storage, the rutin amount underwent a decrease, particularly evident just for HAD and MWD, while FD pollen was able to preserve the highest rutin levels after both three and six months.

### 2.2. Influence of Plant Species, Drying Treatment, and Shelf Life on the Content of Total and Free Amino Acids and Proline

The content of total and free amino acids and total and free proline was highest in ivy pollen (Table 2). Fresh chestnut pollen contained about 50% of the total amino acids of ivy pollen. However, its levels increased in all dehydrated samples, particularly in MWD ones (+57% if compared to the fresh sample). All dehydration treatments reduced the total amino acid content of ivy pollen, which was particularly affected by FD (−25%), this latter being the worst treatment also for willow pollen (−17%) At the end of the storage period, all species of bee-collected pollen, and for any dehydration treatment showed an increased content of total amino acids as compared to the respective treatment at t0.

All the dehydration treatments positively affected the content of the total proline of chestnut pollen—FD was the least effective. Proline level was reduced in ivy pollen in all experiments, whereas HAD and FD induced the highest decrease at t0 (−19% and −25%, respectively). In willow pollen, total proline underwent a marked increase following HAD (+80%), while the other two treatments had a negative impact on this parameter (about −12% and −18% in MWD and FD pollen, respectively). At the end of the storage, all dehydrated samples of chestnut pollen contained significantly less proline if compared to the respective samples at t0, though still higher than the value of fresh pollen. An opposite trend was observed for ivy pollen, whose proline level increased during storage in all dehydrated samples. Both FD and MWD samples of willow pollen, at the end of the storage, contained more proline than at t0 (+10% and +15%, respectively), while the HAD sample underwent a reduction in total proline content during storage (−16% at t6).

The content of free amino acids of chestnut pollen increased following HAD (+17%), while it was negatively affected by FD (−12%) and MWD (−8%). Storage generally led to a significant increase in free amino acids after six months, particularly for FD and MWD samples (+25% and +20%, respectively, as compared to t0).

Ivy pollen underwent a depletion of free amino acids following any kind of dehydration, though MWD was slightly more efficient in preserving their levels. Storage negatively influenced the content of free amino acids after three months almost equally following the three dehydration methods (decrease ranging from −15% to −17%), with no further decrease at t6.

The level of free amino acids of willow pollen was unaffected by FD but underwent a significant decrease following HAD (−17%) and, even more, following MWD (−29%). However, at the end of the storage period, free amino acids were generally more concentrated than at t0 (HAD +18%, MWD +23%) and t3 (HAD + 23%, MWD +50%).

The effect of the dehydration treatments on the content of free proline differed among the studied pollen species. In chestnut pollen, free proline increased following HAD (+11%) while it decreased in FD (−15%) and MWD (−8%) samples. All drying methods reduced the levels of free proline of ivy pollen (up to −24% following FD), though MWD was able to better preserve this amino acid than the other two treatments. However, this dehydration tool was the least effective for willow pollen (−33%), whose free proline, following HAD, remained as concentrated as in fresh pollen. Storage induced a decrease in free proline of chestnut and ivy pollen starting from three months of conservation. In chestnut pollen, such a decrease was particularly evident for HAD samples (−35% at t3). Conversely, the free proline of willow pollen showed a storage-dependent increase after six months of storage (+32%, +25% and +36% following HAD, FD and MWD, respectively, as compared to t0 samples).

### 2.3. Multivariate Analysis of Pollen Components

The complexity of the effects observed between the various samples (Table 1 and Table 2) has led to an intricate interpretation of the results observed, regarding the content of polyphenols, flavonoids, and amino acids. The most effective treatment to improve the shelf-life of the pollen was proven to be the most complicated to interpret. To further investigate the different interactions observed in the samples, the data of polyphenols, flavonoids, and amino acids (total and free) underwent a canonical discriminant analysis to estimate the levels of discrimination between the different groups of pollens.

The canonical discriminant analysis (CDA) was applied to all the components analyzed in the present study, comprising the three pollen species: polyphenols, flavonoids, rutin, total and free amino acids, and total and free proline. Nine canonical functions (CAN) were obtained, then the first two were selected for the subsequent analysis since they explained more than 80% of all observed variance (Table 3).

**Figure 1:**
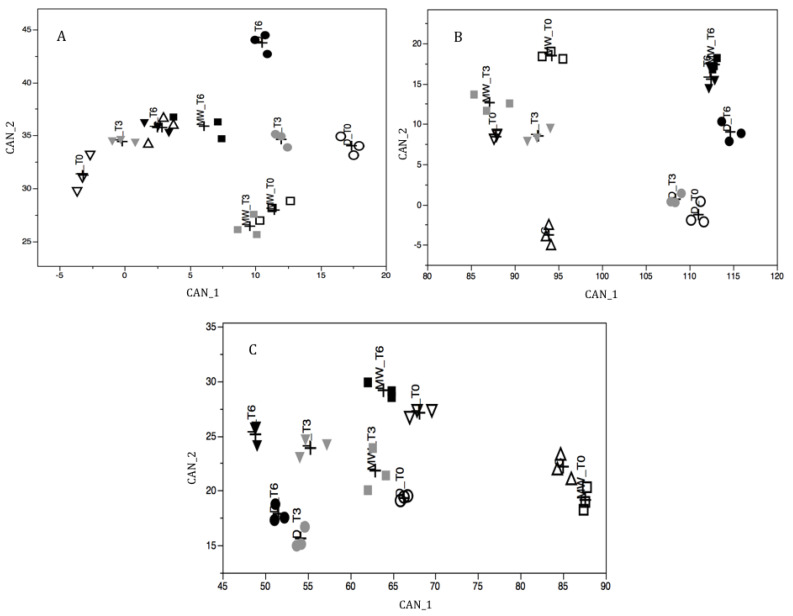
Graph of the canonical function (CAN) for willow (**A**), chestnut (**B**) and ivy (**C**) pollen. Triangles indicate the control, circles indicate hot air drying (HAD), triangles with downward tip indicate freeze-drying (FD), squares indicate microwave-assisted drying (MWD); white symbols indicate t0, grey symbols indicate t3, and black symbols indicate t6.

The extracted CAN significantly discriminated the ten groups (*p*-value Hotelling’s *t*-test *p* < 0.0001) of all the three species of pollen as demonstrated in Figure 1. Based on these results, polyphenols, flavonoids, and rutin showed an opposite effect in comparison to total and free amino acids. Polyphenols, flavonoids, and rutin were associated with FD, while total and free amino acids showed an association with HAD. This result was supported by the higher correlation of FD samples with CAN_1 (negative values) for willow and chestnut pollen and with CAN_1 (positive values) for ivy pollen. For all types of bee-collected pollen, MWD showed intermediate values.

## 3. Discussion

Honeybee-collected pollen has great nutraceutical potential, which is rarely explored in comparison to other bee products [13,16]. The nutraceutical properties of bee-collected pollen are deeply linked to its botanical origin, but dehydration methods, storage conditions and duration influence qualitative markers as well. We previously outlined that chestnut pollen was characterized by a higher level of omega-6 fatty acids, while willow pollen showed a higher concentration of omega-3 fatty acids and carotenoids. FD did not affect the pollen lipid profile, while MWD led to a reduction in tocopherols in bee-collected pollen [16]. Irrespective of microwave power and treatment time, phenol and flavonoid content did not vary over untreated fresh chestnut pollen. Similarly, rutin content was unaffected by the MWD [7]. In the same pollen, the rutin level was affected by FD in a time-dependent manner. However, the free proline to free amino acid ratio was <80%, and the free amino acid to total amino acid ratio was not altered, indicating that FD did not significantly impact the nutritional value of chestnut pollen [11].

According to Komosinska-Vassev et al. [32], pollen can be considered as an excellent source of polyphenols and flavonoids, considering its average content of about 1.6 g/100 g and 1.4 g/100 g, respectively. In our samples, the level of these compounds is much higher even for ivy pollen, which was the poorest source of phenolics and flavonoids as compared to chestnut and willow pollen. Other authors [33], however, detected up to 5 g/100 g of flavonols, a flavonoid subclass. The biosynthesis of phenolic compounds is highly dependent on environmental parameters such as light, temperature, humidity, as well as on the presence of biotic and abiotic stressors. Moreover, intraspecific variability can exist among the different cultivars and ecotypes of the same species, which contributes to explaining the differences observed in pollen collected by bees from the same species (e.g., chestnut) growing in different geographical areas.**** The phenolic concentration of our ivy pollen (always higher than 6 g/100 g in both fresh and dehydrated samples at t0) was also a slightly higher than the amount of methanol soluble phenolics of ivy bee-collected pollen (45.73 g GAE kg^−1^ dry weight) reported by Di Cagno et al. [31]. Such a difference is probably due to the different climatic conditions linked to their geographic origin (Tuscany vs. Apulia) and harvesting year (2015 vs. 2016). The botanical origin of bee pollen influenced the response of the polyphenols to the different drying techniques and their preservation during storage. Chestnut and willow pollen were more sensitive to conventional dehydration, which induced the highest loss of phenolics and flavonoids, though the other treatments also had a negative impact on willow pollen. Conversely, MWD and lyophilization led to an increase in these bioactive compounds in chestnut and ivy pollen, respectively, probably due to a treatment-dependent release of the insoluble fraction of polyphenols. Similar to our finding, Dias et al., [34] reported a better phenolic and flavonoid preservation following lyophilization than air drying in nine types of bee pollen. De-Melo et al., [18] studied the quality of *Eucalyptus* (Myrtaceae) and *Eupatorium* (Asteraceae) bee-collected pollen exposed to different drying processes. In this study, HAD induced the highest phenolic loss, as a probable consequence of the activity of polyphenol oxidase and peroxidases, attributed by the authors to the enzyme release following the frozen-thawing process preceding oven drying. A positive correlation between phenolic content and the antioxidant activity was also noted.

The nutritional and nutraceutical quality of pollen, as of any other food or food supplement, is undoubtedly subjected to storage-dependent decrease. In our research, during storage a progressive decrease in phenolics and flavonoids was observed in chestnut and willow pollen, but not in ivy pollen. We hypothesize that the high proline content of this sample could protect the other biomolecules from oxidation during drying, as usually occurs in drought-stressed plants [35]. However, despite the different influences of the drying systems on the initial content of phenolics and flavonoid of the three pollens, at the end of the storage period, independently from the botanical origin, HAD was always the less efficient treatment, while MWD proved to be a very efficient dehydration method for any pollen.

Rutin is considered a marker of pollen quality for the European market [30]. Chestnut pollen contained the minimum amount of rutin (20 mg/100 g) required to meet the European standards [30], while this parameter was below such a requirement in willow and ivy pollen, both fresh and subjected to the different dehydration treatments. Interestingly, however, fresh ivy pollen contained the highest amounts of total and free amino acids and proline among the three species tested. Among the three species, ivy is the last to bloom and the particularly high amino acid content of its pollen is a precious resource, since pollen represents the main protein source for honeybees, and collection of protein- (or amino acid-) rich pollen in the late season is pivotal for the colony survival [36,37]. The presence of amino acids, together with other biomolecules, is considered responsible for the nutritional value of pollen as a food integrator for children and recovering adults, particularly to promote weight gain and improve the physical and mental fitness [32]. Therefore, though ivy pollen does not meet the quality requirements related to phenolic content, the high level of amino acids still makes it a valuable food product.

As reported by Canale et al. [7], the content of free amino acids should be at least 2% to meet the quality standards required by the European market for bee-collected pollen. In addition to ivy pollen, which largely overwhelms this requirement, also chestnut and willow pollen respect this condition, either fresh and dehydrated, the only exception being willow pollen subjected to MWD at t0 and after three months of storage. Dominguez-Valhondo et al. [38] observed a decrease in the content of free amino acids of multifloral pollen during the drying processes, more severe in the hot-air-dried samples than the ones that have been lyophilized. As reported by Serra Bonvehí and Escolà Jordà [39], free amino acids are sensitive to dehydration temperature and duration. In the present research, the preservation of good amounts of free aminoacids also in HAD samples may depend on the lower dehydration temperature (32 °C) if compared to that used by Dominguez-Valhondo [38] (76 °C).

Proline is the main free amino acid in bee-collected pollen. In our study, its content decreased following any drying process, except for conventional HAD in chestnut and willow pollen samples. However, the total proline of dehydrated chestnut pollen accumulated at higher levels compared to a fresh sample, particularly after HAD and MWD. The same trend was observed also for HAD willow pollen. Of note, the mechanisms at the basis of the observed variation are still unknown. Earlier, Kanar and Mazi [40] observed only slight, not significant, decreases in proline content of multifloral fresh bee pollen dehydrated by different techniques, such as conventional HAD and lyophilization. However, vacuum drying (100 mbar at 65 °C), microwave drying at 600 and 900 W, and assisted microwave drying (675 mbar) at 900 W induced a significant loss of proline. A similar decreasing trend was reported by Dominguez-Valhondo [38] in multifloral pollen after FD and HAD (70 °C for 7 h). Though, generally, proline content was negatively affected by dehydration, differences in drying parameters, such as duration, temperature and microwave power, are probably at the basis of the specific evidences reported in literature. Moreover, since proline concentration depends on the equilibrium between its degradation and production at the expense of glutamic acid, a different influence of the dehydration methods on gluatamic dehydrogenase activity cannot be excluded. Another marker related to pollen quality is represented by the proline index (free proline to free amino acids ratio). Proline is the main aminoacid of dehydrated pollen, and its production from glutamic acid during dehydration depends on the presence and activity of glutamic dehydrogenase. Since prolonged drying and/or excessive temperatures induce a decrease in free amino acid levels, the proline index increases in low-quality pollens [39]. According to these authors, this index should be below 80%. Fresh willow pollen presents the lowest proline index (38.5%), while the other two pollens displayed relatively high indexes (about 65% and 72%, chestnut, and ivy, respectively). However, this index never overcame the limit because of the different dehydration treatments and storage. Accordingly, it can be concluded that both FD and MWD techniques are efficient in preserving the amino acids-related quality of bee pollen up to six months of storage.

## 4. Materials and Methods

### 4.1. Bee-Collected Pollen

Bee-collected chestnut (*C. sativa*) pollen was harvested by a beekeeper in July 2015 in chestnut growing sites (44°06′22.7″ N 10°24′02.7″ E, Castelnuovo Garfagnana, Lucca, Central Italy), using a pollen trap (A. Metalori, Italy). Willow and ivy pollens were harvested by a beekeeper in April and September 2015, respectively, in *S. alba* orchards and *H. helix* growing sites, both located in Massa Macinaia (43°47′45.6″ N 10°32′03.2″ E, Capannori, Lucca, Central Italy), with the Metalori pollen trap mentioned above.

All pollen samples were immediately frozen and transferred to the laboratories for further conditioning. For all pollens, their monofloral origin was identified by color and light microscopy examination (400×) [41]. All pollens were identified through comparison with pollen atlas databases [12,41,42]. Post-conditioning, all analytical results were compared with the fresh untreated pollen sample, 4 pollen samples per species (i.e., chestnut, willow, and ivy) were collected and each sample was analyzed in triplicate to reduce the individual variability. Moreover, 24 samples were collected to evaluate the effect of post-harvest conditioning over time: (i) 6 untreated samples (control), (ii) 6 conventionally dried samples, (iii) 6 freeze-dried samples, and (iv) 6 microwave-dried samples. In each group, 2 samples were stored for different periods (i.e., 0, 3, and 6 months, at 23 °C in airtight containers) and then analyzed to assess the shelf-life impact.

### 4.2. Conventional Drying

Commercially dried samples were obtained treating bee-collected pollen at 32 °C for 24 h in the NTW 100 cool-air dryer (Northwest Technology, Boves, Cuneo, Italy, http://www.northwest-technology.com/), as recently reported by Conte et al. [16]. Residual water in the dried sample was 7%, according to the Italian commercial standards for commercializing bee-collected pollen for human consumption. NTW 100 has been selected as a conventional drying tool since is widely used by Italian pollen producers to treat bee-collected pollen, allowing to dry 100 kg of fresh pollen each cycle (i.e., in 24 h). 

### 4.3. Freeze-Drying

FD was carried out on all treated and untreated pollens following the recent method detailed by Conte et al. [16]. Briefly, a lyophilizer Heto PowerDry^®^ LL1500 with 4 manifolds connecting to 100 mL flasks filled with 70 g of fresh pollen (earlier stored at −20 °C) was used. Temperature in the condensation chamber was −115 °C, with a full vacuum. Heat exchange in Heto PowerDry^®^ LL1500 was achieved by convection with the temperature of the test room, which was continuously conditioned at 25 °C. The maximum temperature reached by the sample during the process was 25 °C. To carry out pollen FD, full vacuum was created in the flasks. Each pollen sample was inserted into the chamber, its initial weight was recorded and then the sample was treated for 9 h. The exposure period was determined by preliminary tests followed by thermogravimetric analysis (TGA). The final weight was determined and the samples were sealed and stored at −20° C until the analyses [16]. The liquid inside the condensation chamber was sampled, sealed, and stored at a temperature of −20° C for further analysis. TGA (TGA Pyris 1 Perkin Elmer (Waltham, MA, USA)) conducted at 120 °C showed that the residual water content was 6.0%, 6.3%, and 7.5% for chestnut, willow, and ivy pollen, respectively.

### 4.4. Microwave-Assisted Drying

MWD was conducted according to Canale et al., [7] and Conte et al. [16]. MW was conducted at 50 mbar; MW power was 150 W; the exposure time was 30 min for all pollens, except for ivy pollen, which was treated for 75 min. Preliminary observations showed that a 40-min treatment was not sufficient for ivy, leading to 12.5% of residual water content. After MW drying, the pollen was weighed and had its temperature measured with a K-type thermocouple. Then, the sample was moved to an airtight container at −20 °C until analysis.

TGA conducted at 120 °C showed that the residual water content was 6.4%, 10.3%, and 8.2% for chestnut, willow, and ivy pollen, respectively.

### 4.5. Extraction and Quantification of Phenolics and Flavonoids

The extraction was performed in triplicate as reported by Canale et al., [7] with minor changes by Ranieri et al. [11]. Briefly, 0.5 g of bee pollen was extracted with 80% methanol aqueous solution by ultrasonic bath sonication (30 min) followed by 30 min magnetic stirring at 4 °C. The supernatant was recovered by 15 min centrifugation at 14,000× *g* (4 °C). The pellet was re-extracted twice without the sonication step and the pooled supernatants were filtered (0.45 µm Minisart filters, Sartorius Stedim Biotech, Goettingen, Germany).

Phenolics were quantified with the Folin–Ciocalteu colorimetric method [43] based on phenol oxidation by two strong inorganic oxidants (phosphotungstic and phosphomolybdic acids) in an alkaline medium. The absorbance of the reaction mix containing Folin–Ciocalteu reagent (0.125 mL), distilled water (1.85 mL), 20% Na_2_CO_3_ (0.5 mL), and the extract (25 µL) was read at 750 nm after 30 min incubation. Phenolic concentration was expressed as mg of gallic acid equivalents (GAE) g^−1^ dry weight (DW).

Flavonoids were determined according to Kim et al., [44] and expressed as mg of catechin equivalents g^−1^ DW. Briefly, 60 µL of 5%NaNO_2_, 40 µL of 10% AlCl_3_, 400 µL of 1 M NaOH, 200 µL of distilled water and 100 µL of extract were mixed, and the absorbance was recorded at 510 nm.

All reagents were purchased from Sigma Aldrich Chemical Co. (St. Louis, MO, USA).

### 4.6. Rutin Quantification by HPLC Analysis

**Figure 2:**
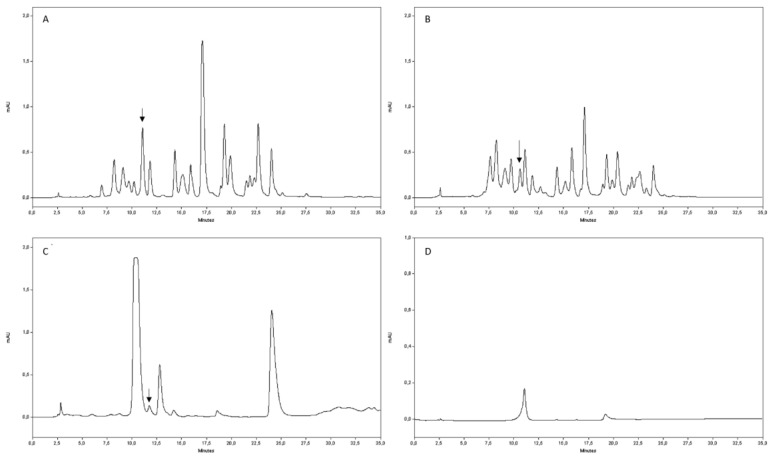
Chromatograms of phenolic extracts of bee-collected pollen: (**A**) chestnut, (**B**) willow, and (**C**) ivy. (**D**) Rutin standard. Arrows indicate rutin.

Rutin was quantified by a Spectra System P4000 HPLC equipped with a UV 6000 LP photodiode array detector (Thermo Fisher Scientific, Waltham, MA, USA) using a Prodigy LC-18 RP column (5 µm particle size, 250 × 4.6 mm, Phenomenex Italia, Castel Maggiore, Bologna, Italy) (Figure 2). Elution was performed at a flow rate of 1 mL min^−1^ according to the following gradient: 0–5 min 90% solvent A (5% formic acid in water) 5–20 min 90–70% solvent A, 20–28 min 70–10% solvent A, 28–35 min 10–90% solvent A, followed by 5 min re-equilibration in the initial condition before the next injection. Solvent B was 5% formic acid in methanol. The detector was set at 340 nm. The commercial standard of rutin (Sigma Aldrich Chemical Co., St. Louis, MO, USA) was used to obtain the calibration curve (0–100 ppm). Solvents were of HPLC grade; they were purchased from Sigma Aldrich Chemical Co. (St. Louis, MO, USA).

### 4.7. Extraction and Quantification of Free and Total Amino Acids

The analysis was carried out as previously reported by Canale et al. [7]. Briefly, 0.1 g bee pollen was ground with 80% ethanol, followed by 5 min sonication. The supernatant was recovered by 15 min centrifugation at 12,100 g and the pellet was re-extracted. The pooled supernatants were vacuum dried, dissolved in MilliQ water, filtered with 0.45 µm Minisart filters (Sartorius Stedim Biotech, Goettingen, Germany), and used for determination of free proline and free amino acids.

Total amino acids were extracted by digesting 0.1 g of bee pollen with 6 N HCl at 110 °C for 24 h. The extracts were treated as described above for free amino acids.

Free and total amino acid concentration was quantified according to Canale et al. [7]. Extract (0.1 mL) was added to 1 mL 0.2 M Na citrate buffer, 8 mM SnCl_2_ and 1 mL of 4% ninhydrin solution in ethylene glycol. The mix was incubated at 100 °C for 20 min and after cooling at room temperature, 5 mL of 50% isopropanol was added. Absorbance was recorded at 570 nm. The amino acid concentration was calculated using a calibration curve of leucine (3–30 µg). Since proline is not detectable by this method, the concentration of free and total amino acids was calculated by summing proline concentration determined as reported below. All data are expressed as mg g^−1^ DW. All reagents were purchased from Sigma Aldrich Chemical Co. (St. Louis, MO, USA).

### 4.8. Quantification of Free and Total Proline

Proline concentration was determined following the method reported by Canale et al., [7] and Ranieri et al., [11], using 1% ninhydrin (Sigma Aldrich Chemical Co., St. Louis, MO, USA) in 60 % acetic acid. Samples (0.5 mL) were incubated with 2 mL ninhydrin at 100°C for 1 h and, after the addition of 5 mL toluene to extract the chromophore, absorbance of the upper phase was read at 520 nm. Proline was quantified using a calibration curve of 3–30 µg proline.

### 4.9. Data Analysis

Data on rutin, polyphenols, flavonoids, amino acid, and proline content were analyzed by the following full factorial linear model, using the software JMP 9 (SAS, SAS Institute Inc., Cary, NC, USA):

y_ijzk_ = µ + P_i_ + T_j_ + S_z_ + P_i_ × T_j_ + T_j_ × S_z_ + P_i_ × S_z_ + P_i_ × T_j_ × S_z_ + ε_ijzk_

where: y_ijzk_ = rutin, polyphenols, flavonoids, amino acid, and proline content; P_i_ = fixed effect of the i-th pollen species (willow, chestnut, and ivy); T_j_ = fixed effect j-the treatment (fresh, HAD, FD, MWD); S_z_ = fixed effect of the z-th shelf-life time (t0, t3, t6).

The Tukey’s HSD test with a 95% confidence level was applied to evaluate the effect of the different treatments. We used JMP software (SAS Institute Inc., Cary, NC, USA). All analyses were replicated on three independent samples.

Canonical discriminant analysis (CDA) was used to investigate for possible effects of the treatment on pollen composition during the shelf-life. For this reason, the following components were used: rutin, polyphenols, flavonoids, total proline and amino acids, free proline, and amino acids. These components are a useful indicator of antioxidant effect and pollen degradation.

The CDA is a dimension-reduction technique that performs both univariate and multivariate one-way analysis, thanks to the principal component analysis and canonical correlation. Given a classification character and several interval variables, CDA derives a set of new variables called canonical functions (CAN), which are linear combinations of the original interval variables, as reported in the following equation:

CAN = *d*_1_*X*_1_ + *d*_2_*X*_2_ + …+ *d_n_X_n_*

where *d_i_* are the canonical coefficients (CC) that indicate the contribution of each variable in composing the CAN, and *X_i_* are the scores of the n original variables.

CANs summarize the between-group variations, highlighting their differences. In general, if k groups are involved in the study, k-1 CANs are extracted. The effective separation between groups was assessed by using the Mahalanobis distance and the corresponding Hotelling’s T-square test [45].

Subsequently, the ability of CAN to assign each pollen to the twelve groups was calculated as the percent of correct assignment using discriminant analysis (DA) [46]. Practically, CAN is applied to each pollen and a discriminant score is obtained. A sample is assigned to one of the 12 groups if its discriminant score is lower than the cutoff value obtained by calculating the weighted mean distance among group centroids [46].

## 5. Conclusions

The chemical composition of the bee-collected pollen was strictly dependent on its botanical origin. Ivy pollen contained the highest amount of total and free amino acids and proline, while chestnut pollen was the richest source of polyphenols. Our research indicates that the botanical origin of pollen also influenced its sensitivity to the different dehydration techniques and the preservation of its quality during storage. Since polyphenols and amino acids of a particular pollen species are often differently influenced by the same dehydration method, the choice of the best dehydration technique should be decided based on the quality trait to be preferentially preserved.

## Figures and Tables

**Table 1:** Effect of species of pollen, treatment, and shelf-life on polyphenols and flavonoids content.

Time	Treatment	Species			Factors ^†^	*p*-Value	Standard Error (SE)
		Chestnut	Ivy	Willow			
Phenolics (mg GAE g^−1^ DW)				
t0	Fresh	24.77 ^AY1^	6.37 ^CY1^	21.10 ^BX1^	P	***	0.22
	HAD	23.27 ^AZ1^	6.24 ^CY1^	17.29 ^BZ1^	T	***	
	FD	24.37 ^AY1^	6.83 ^CX1^	18.26 ^BY1^	S	***	
	MWD	30.50 ^AX1^	6.20 ^CY1^	19.44 ^BY1^	P × T	**	
					P × S	***	
t3	HAD	21.68 ^AZ2^	5.27 ^CZ1^	17.60 ^BX2^	S × T	ns	
	FD	22.87 ^AY2^	6.89 ^CY1^	16.65 ^BY2^	P × T × S	***	
	MWD	25.10 ^AX2^	7.68 ^CX1^	16.78 ^BY2^			
t6	HAD	20.61 ^AY3^	6.20 ^CZ1^	14.28 ^BY3^			
	FD	23.05 ^AX3^	7.75 ^CY1^	16.11 ^BX3^			
	MWD	23.28 ^AX3^	9.78 ^CX1^	16.41 ^BX3^			
Flavonoids (mg of catechin equivalents g^−1^ DW)				
t0	Fresh	10.13 ^Ab1^	1.54 ^Cb1^	8.20 ^Ba1^	P	***	0.10
	HAD	7.25 ^Ac1^	1.87 ^Cb1^	5.86 ^Bc1^	T	***	
	FD	8.53 ^Ac1^	2.00 ^Ca1^	7.22 ^Bb1^	S	***	
	MWD	11.66 ^Aa1^	1.68 ^Cb1^	6.93 ^Bb1^	P × T	***	
					P × S	***	
t3	HAD	8.13 ^Ab2^	1.77 ^Cb1^	6.10 ^Bb2^	S × T	ns	
	FD	9.19 ^Aa2^	2.12 ^Ca1^	6.53 ^Ba2^	P × T × S	***	
	MWD	9.72 ^Aa2^	1.84 ^Ca1^	6.62 ^Ba2^			
t6	HAD	7.05 ^Ac2^	1.87 ^Cb1^	4.57 ^Bc3^			
	FD	7.91 ^Ab2^	2.28 ^Ca1^	5.95 ^Bb3^			
	MWD	8.47 ^Aa2^	2.29 ^Ca1^	6.31 ^Ba3^			
Rutin (µg g^−1^ DW)				
t0	Fresh	326.35 ^Ab1^	9.04 ^Ca1^	78.37 ^Bc1^	P	***	3.51
	HAD	242.42 ^Ac1^	3.81 ^Cb1^	138.04 ^Ba1^	T	***	
	FD	316.46 ^Ab1^	10.99 ^Ca1^	94.14 ^Bb1^	S	*	
	MWD	369.29 ^Aa1^	5.81 ^Cb1^	121.67 ^Ba1^	P × T	***	
					P × S	***	
t3	HAD	253.69 ^Ab1^	4.21 ^Ca2^	87.68 ^Bb2^	S × T	*	
	FD	331.09 ^Aa1^	6.53 ^Ca2^	104.00 ^Ba2^	P × T × S	***	
	MWD	324.93 ^Aa1^	5.87 ^Ca2^	56.67 ^Bc2^			
t6	HAD	287.30 ^Ac1^	4.83 ^Ca2^	54.89 ^Bc2^			
	FD	363.09 ^Aa1^	7.27 ^Ca2^	108.68 ^Ba2^			
	MWD	344.20 ^Ab1^	6.17 ^Ca2^	86.59 ^Bb2^			

^A–C^ Indicate significant differences between the species within the row (*p* < 0.01). ^a–c^ Indicate significant difference between the treatment within the columns (*p* < 0.01). ^1–3^ Indicate significant difference between the time within the columns (*p* < 0.01). ^†^ Factors included in the model: P = effect of pollen species; T = effect of treatment; S = effect of shelf-life. DW = dry weight, GAE = gallic acid equivalents. ***, **, * indicate a significant difference (*p* < 0.001, *p* < 0.01, *p* < 0.05, respectively), ns = not significant (*p* > 0.05).

**Table 2:** Effect of species of pollen, treatment, and shelf-life on total and free amino acids and proline.

	Time	Treatment	Species			Factors ^†^	*p*-Value	Standard Error (SE)
			Chestnut	Ivy	Willow			
Total Amino Acid (mg g^−1^ DW)								
	t0	Fresh	183.50 ^Cc2^	363.15 ^Aa2^	259.72 ^Ba2^	P	***	3.83
		HAD	261.14 ^Bb2^	287.85 ^Ab2^	261.22 ^Ba2^	T	***	
		FD	223.60 ^Bc2^	273.92 ^Ac2^	214.93 ^Bc2^	S	***	
		MWD	287.89 ^Aa2^	287.12 ^Ab2^	247.43 ^Bc2^	P × T	**	
						P × S	***	
	t3	HAD	233.15 ^Cb2^	347.75 ^Ab1^	247.84 ^Ba2^	S × T	ns	
		FD	233.15 ^Bb2^	327.47 ^Ac1^	214.66 ^Cb2^	P × T × S	***	
		MWD	242.71 ^Ba2^	402.28 ^Aa1^	215.16 ^Cb2^			
	t6	HAD	286.18 ^CY1^	358.16 ^Ab1^	299.59 ^Ba1^			
		FD	300.37 ^Ba1^	345.66 ^Ab1^	264.71 ^Cb1^			
		MWD	310.98 ^Ba1^	393.61 ^Aa1^	276.33 ^Cb1^			
Total Proline (mg g^−1^ DW)								
	t0	Fresh	28.99 ^Bc1^	95.07 ^Aa2^	29.03 ^Bb2^	P	***	0.79
		HAD	57.02 ^Ba1^	77.05 ^Ac2^	52.13 ^Ca2^	T	***	
		FD	39.31 ^Bc1^	71.24 ^Ac2^	23.80 ^Cc2^	S	*	
		MWD	61.06 ^Ba1^	84.60 ^Ab2^	25.58 ^Cc2^	P × T	***	
						P × S	***	
	t3	HAD	36.23 ^Cb1^	84.64 ^Aa1^	41.08 ^Ba1^	S × T	**	
		FD	36.23 ^Bb1^	78.66 ^Ab1^	22.91 ^Cc1^	P × T × S	***	
		MWD	45.40 ^Ba1^	85.72 ^Aa1^	27.63 ^Cb1^			
	t6	HAD	36.75 ^Cb2^	76.75 ^Ac1^	44.01 ^Ba1^			
		FD	37.79 ^Bb2^	80.78 ^Ab1^	26.21 ^Cc1^			
		MWD	40.74 ^Ba2^	95.82 ^Aa1^	29.43 ^Cc1^			
Free Amino Acid (mg g^−1^ DW)								
	t0	Fresh	25.01 ^Cb2^	87.44 ^Aa1^	27.91 ^Ba2^	P	***	0.55
		HAD	29.31 ^Ba2^	69.74 ^Ac1^	23.17 ^Cb2^	Tr	ns	
		FD	21.93 ^Cc2^	71.85 ^Ac1^	28.40 ^Ba2^	Ti	***	
		MWD	22.97 ^Bc2^	79.26 ^Ab1^	19.90 ^Cc2^	P × Tr	***	
						P × Ti	***	
	t3	HAD	29.31 ^Ba2^	58.00 ^Ac2^	22.33 ^Ca2^	Ti × Tr	ns	
		FD	23.44 ^Bb2^	60.45 ^Ab2^	22.37 ^Ba2^	P × Tr × Ti	***	
		MWD	21.63 ^Bb2^	67.10 ^Aa2^	17.60 ^Cb2^			
	t6	HAD	28.87 ^Ba1^	55.16 ^Aa2^	27.40 ^Ba1^			
		FD	27.42 ^Bb1^	53.41 ^Aa2^	25.52 ^Cb1^			
		MWD	27.57 ^Bb1^	64.86 ^Aa2^	26.47 ^Ba1^			
Free Proline (mg g^−1^ DW)								
	t0	Fresh	16.24 ^Bb1^	63.47 ^Aa1^	10.75 ^Ca2^	P	***	0.38
		HAD	17.95 ^Ba1^	50.88 ^Ac1^	11.04 ^Ca2^	T	*	
		FD	13.88 ^Bc1^	48.37 ^Ac1^	9.17 ^Cb2^	S	***	
		MWD	14.97 ^Bc1^	55.24 ^Ab1^	7.15 ^Cc2^	P × T	***	
						P × S	***	
	t3	HAD	11.58 ^Bb2^	40.43 ^Ab2^	11.73 ^Bb2^	S × T	ns	
		FD	14.04^Ba2^	36.96^Ac2^	13.56^Ba2^	P × T × S	***	
		MWD	13.42 ^Ba2^	42.39 ^Aa2^	7.35 ^Cc2^			
	t6	HAD	15.13 ^Ba2^	37.37 ^Ab2^	14.56 ^Ba1^			
		FD	14.91 ^Bb2^	33.61 ^Ac2^	11.42 ^Cb1^			
		MWD	14.31 ^Bb2^	41.87 ^Aa2^	9.72 ^Cc1^			

^A–C^ Indicate significant differences between the species within the row (*p* < 0.01). ^a–c^ Indicate significant difference between the treatment within the columns (*p* < 0.01). ^1–3^ Indicate significant difference between the time within the columns (*p* < 0.01). ^†^ Factors included in the model: P = effect of pollen species; T = effect of treatment; S = effect of shelf-life. DW = dry weight. ***, **, * indicate a significant difference (*p* < 0.001, *p* < 0.01, *p* < 0.05, respectively), ns = not significant (*p* > 0.05).

**Table 3:** Correlations between canonical (CAN) and original variables.

	CAN_1	CAN_2
*Willow*		
Polyphenol	−0.17	−0.34
Flavonoids	**−0.45**	−0.38
Rutin	0.06	−0.07
Total Amino Acids	**0.40**	**0.71**
Total Proline	**0.78**	**0.41**
Free Amino Acids	**0.46**	**0.65**
Free Proline	−0.06	**0.87**
Variance Explained (%)	49.38	31.99
Cumulative Variance (%)	49.38	81.37
*Chestnut*		
Polyphenol	**−0.47**	0.32
Flavonoids	**−0.63**	0.24
Rutin	**−0.40**	**0.69**
Total Amino Acids	**0.63**	**0.74**
Total Proline	−0.03	0.31
Free Amino Acids	**0.94**	−0.31
Free Proline	0.18	−0.23
Variance Explained (%)	61.07	29.53
Cumulative Variance (%)	61.07	90.60
*Ivy*		
Polyphenol	−0.14	**0.82**
Flavonoids	**−0.57**	**0.62**
Rutin	0.25	**0.67**
Total Amino Acids	0.05	−0.04
Total Proline	**0.47**	0.07
Free Amino Acids	**0.99**	−0.01
Free Proline	**0.97**	−0.09
Variance Explained (%)	84.46	8.83
Cumulative Variance (%)	84.46	93.29

Values in bold represent high correlation (>0.4 in absolute value).
